# Brain abscess and bronchopneumonia caused by *Acinetobacter baumannii* in a 2-year-old female sheep

**DOI:** 10.1080/01652176.2018.1489165

**Published:** 2018-10-30

**Authors:** Yesari Eroksuz, Baris Otlu, Hatice Eroksuz, Nafia Canan Gursoy, Zeynep Yerlikaya, Canan Akdeniz Incili, Burak Karabulut, Necati Timurkaan, Mehmet Ozkan Timurkan

**Affiliations:** a Department of Pathology, School of Veterinary Medicine, Firat University, Elazig, Turkey;; b Department of Medical Microbiology, School of Medicine, Inonu University, Malatya, Turkey;; c Department of Microbiology, School of Veterinary Medicine, Firat University, Elazig, Turkey;; d Department of Virology, School of Veterinary Medicine, Ataturk University, Erzurum, Turkey

**Keywords:** Sheep, ovine; *Acinetobacter baumannii*, brain abscess, pneumonia

**Figure 1.**
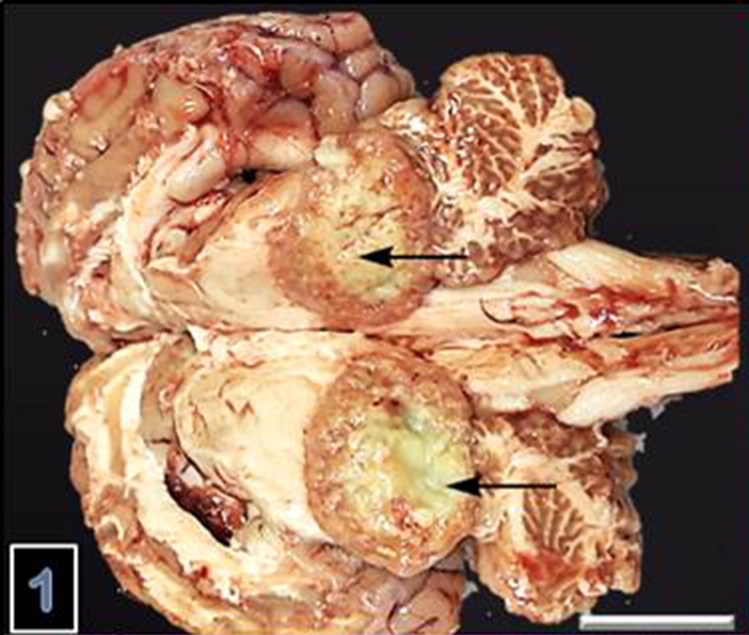
Suppurative, focally extensive encephalitis and abscess in cerebral pelliculi of brain.

**Figure 2.**
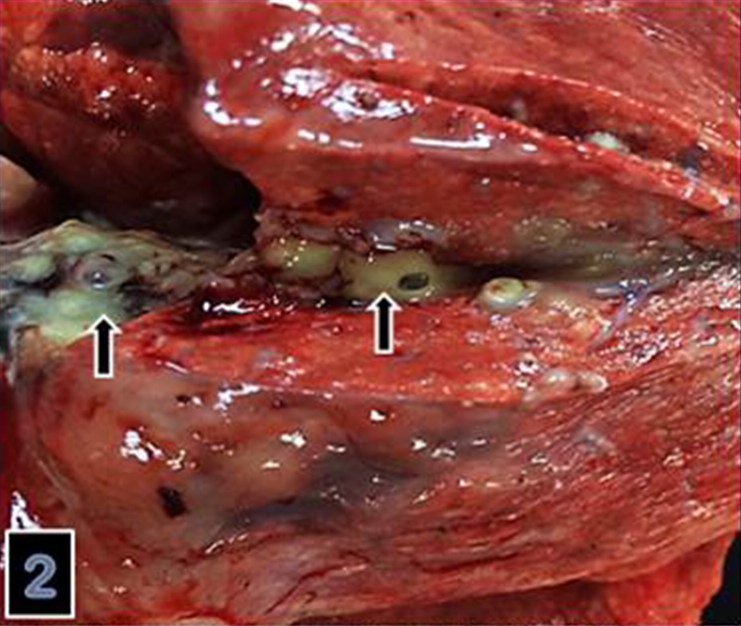
Purulent exudate squizing from bronchial lumina into cut surfaces of the lung.

A 2-year-old, female sheep was admitted for necropsy to Pathology Department, Veterinay Medical Faculty, Firat University with a history of loss of appetite, weight loss, coughing and nervous signs characterized by shaky gait and inability to rise at final stage of illness. There were totally 10 sheep exhibiting similar clinical signs with 8 deads in past 28 days out of 100 sheep in total. The animals had been examined by referring veterinary practitioner prescribing oxytetracycline (Primamycin-LA, Pfizer Inc., New York, NY, USA, at 10 mg/kg BW every 24 h, intramuscularly, for 4 days). At necropsy, the most prominent change was an abscess in brain colliculi and measuring 4 × 4 × 3 cm (Figure 1). The content of abscess was yellow to light greenish in color and had gelatinous consistency with mild liquification in the center. In lungs, there was purulent exudate obstructing and thickening bronchial walls and focal abscess formation on cut surfaces measuring 1–2 mm (Figure 2). There were two hydatit cysts measuring 7–8 cm in diameter in visceral surfaces of the liver and lungs.

Representative samples of all lesions and major organs were fixed in 10% neutral buffered formalin. Samples were paraffin embedded, prepared as 5 μm sections, and stained with hematoxylin and eosin (H&E). Selected tissue sections were stained with Gomori’s methanamine silver nitrate (GMS), Brown-Brenn’s (B-B) method and Giemsa. The bacterial culture material was fixed in 2.50% glutaraldehyde in 0.1 M phosphate buffer (pH 7.3) and then post-fixed in 1.0% osmium tetroxide solution in same buffer solution and then dehydrated in graded ethanol solution and coated with a gold layer (10 nm). The culture material was analyzed using scanning electron microscope (SEM).

**Figure 3.**
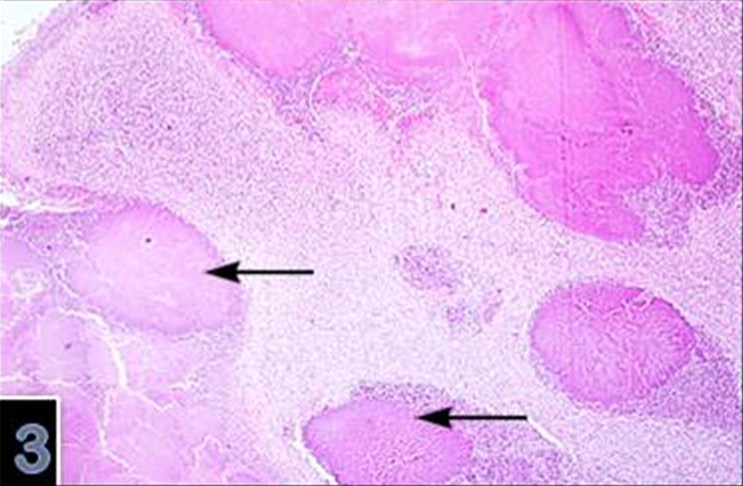
Multifocal-coalesing necrotic foci (arrows) surounded by inflammatory cells in the abscess (magnification 4×).

**Figure 4.**
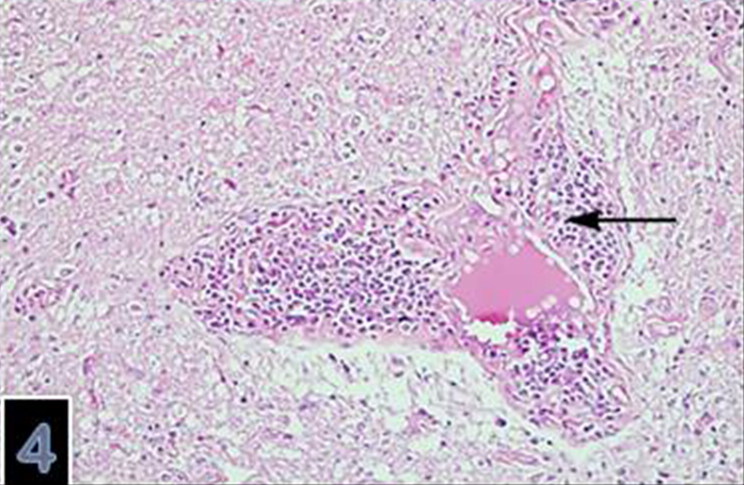
Lympho-histiocytic infiltration in Virchow-Robin Spaces (magnification 10×).

**Figure 5.**
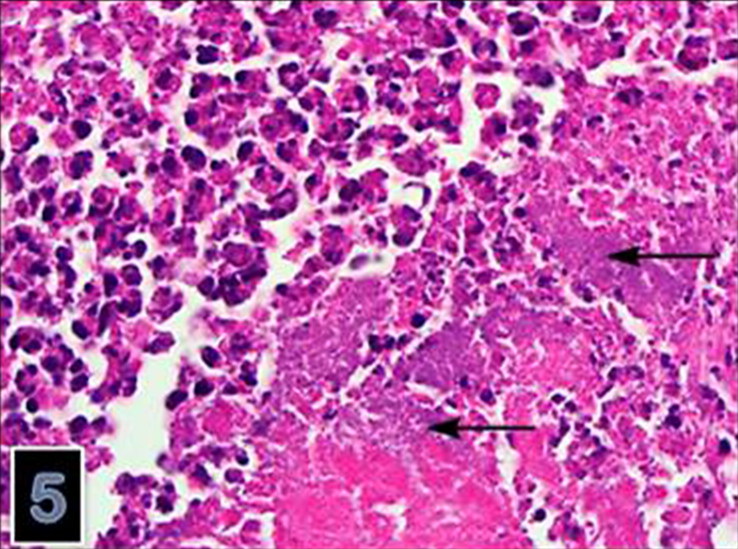
The microorganism consisting of clumbs or aggregates was present in necrotic foci (HE) (magnification 40×).

**Figure 6.**
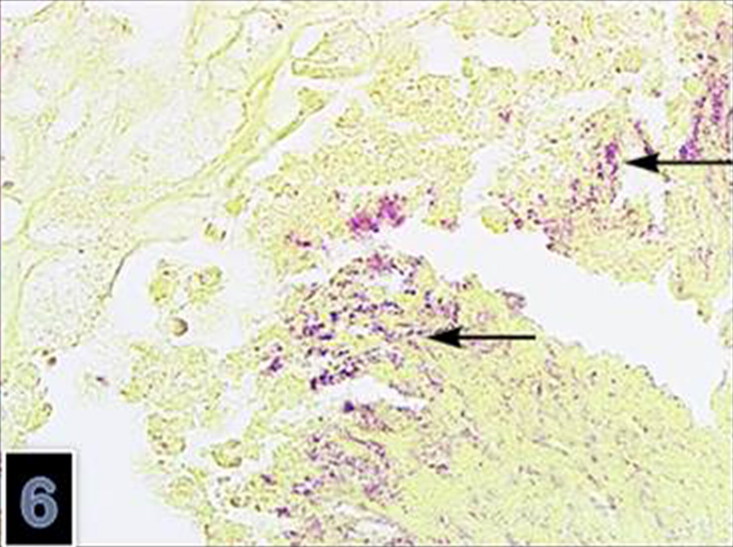
Clumps of the Gram-negative microoorganism (B-B) in the brain (magnification 40×).

**Figure 7.**
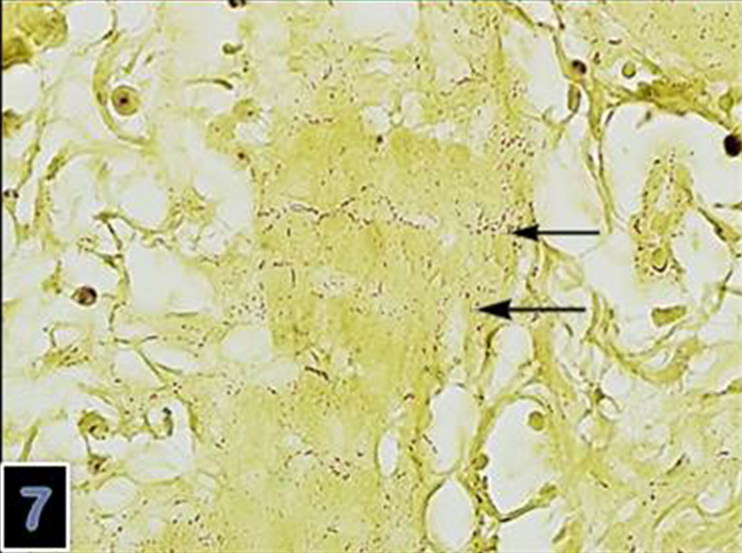
Clumps of the Gram-negative microoorganism (B-B) in the lungs (magnification 40×).

**Figure 8.**
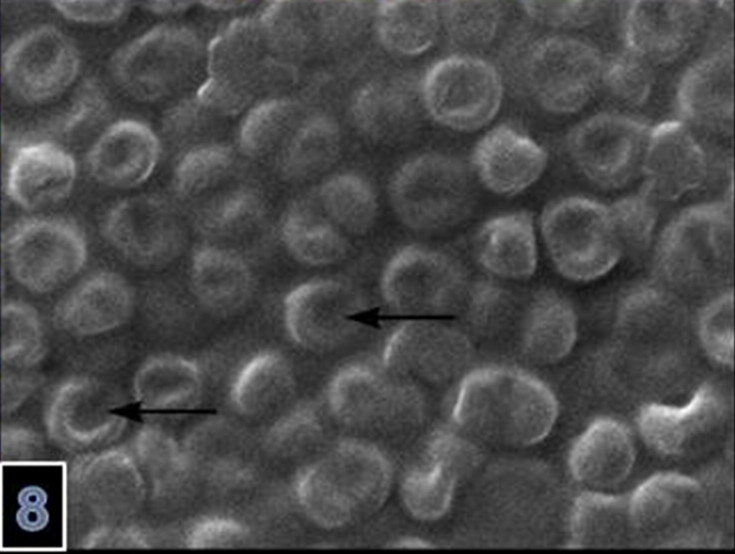
The microorganism is round in appearence with the central region hollowing inward (SEM) (magnification 30,000×).

Microscopically, varying sized necrotic foci with a tendency to coalesce were detected in the brain (Figure 3). There was also lympho-histiocytic infiltration in Virchow-Robin Spaces (Figure 4). Necrotic foci contained central necrosis with cellular debris and clumbs of microorganisms surrounded by dead neutrophils, macrophages and few lymphocytes and plasma cells surrounded by granulation tissue and gliosis. The microorganisms consisting of clumbs or aggregates were present in necrotic foci (Figure 5) and identified as Gram negative (Figures 6 and 7). SEM showed details of the round organisms measuring 525–650 µm in diameter with the central region hollowed inward (Figure 8). In the eyes, there was marked papillary edema at the optic nerve head. Morphologic diagnoses included chronic purulent bronchopneumonia with fibrosis, acute cerebral abscess, echinococcosis and papillary edema of the optic nerve head. Other lesions were unremarkable.

The bacteria isolated from lungs and brain abscess were grown on blood agar base containing 5.0% sterile defibrinated sheep blood at 37.5 °C for 48 h at aerobic conditions. The colonies were mucoid and greyish in color. The bacteria were Gram negative and small, plump rods in shape. Catalase and oxidase reactions were positive and negative, respectively. The microorganism was grown on MacConkey agar, and demonstrated not to metabolize glucose in oxidation and fermantation tests. The bacteria were nonmotile in the motility test.

The colonies were described as *Acinetobacter baumannii* (99.9%) by using Matrix-mediated laser desorption ionization-flight time mass spectrometry (MALDI-TOF MS) (database v2.0, bioMérieux, France) system.

Standard disc-diffusion method was used for the antibiotic susceptibility test and the results were interpreted according to the EUCAST criteria (The European Committee on Antimicrobial Susceptibility (EUCAST)) 2015. Testing breakpoint tables for interpretation of MICs and zone diameters were used according to version 7.1. available at www.eucast.org/clinical_breakpoints (accessed 2018 February 22).

The isolate was susceptible to cefepime, ceftriaxone, imipenem, piperacillin, piperacillin–tazobactam, ceftazidime, aztreonam, cefuroxime, ciprofloxacin, meropenem, amikacin, norfloxacin, cotrimoxazole, colistin, whereas it was resistant to ampicillin, cefoxitin, fosfomycin, ertapenem and amoxicillin clavulanic acid. Among the antibiotics tested, use of cephalosporins and penicillin group drugs such as piperacillin/tazobactam are currently considered to be inappropriate to the classical guidelines due to the highly resistant isolates of *Acinetobacter* spp. in humans and therefore clinical use and routine reporting are not recommended. Our isolate has been shown to be sensitive *in vitro* to these drugs, but clinical use of these drugs is not recommended.

Tissue samples from brain and lung were mechanically sectioned with a Polytron PT 1200 E (Kinematica, Lucerne, Switzerland) tissue mill. Total DNA isolation was then performed using the BioRobot® EZ1 (Qiagen, Hilden, Germany) automated extraction device using the DNA Tissue Kit (Qiagen, Hilden, Germany) as recommended by the manufacturer.

Formalin-fixed paraffin-embedded (FFPE) DNA was isolated using the QIAamp DNA FFPE Tissue kit (Qiagen, Hilden, Germany) according to the manufacturer's recommendation. For deparaffinization, the sections taken into microcentrifuge tubes; 1 ml xylene was added, vortexed for 10 s, and the samples were centrifuged for 2 min at 13,000 × g. After centrifugation, the cells were washed twice with 1 ml of ethanol (100%) to remove the xylene from the pellet and the alcohol was removed by leaving the openings in the biosafety chamber for 10 min. After this step, DNA isolation was carried out using a column-based DNA isolation kit (DNA mini kit, Qiagen, Germany).

Following the DNA isolation from the direct tissue sample and FFPE samples, a partial 16S rDNA sequence was constructed using primers p8FPL 5′AGT TTG ATC ATG GCT CAG-3′ and p806R 5′-GGA CTA CCA GGG TAT CTA AT-3′. An approximately 800 base-pair 16S rDNA region was inserted in both directions using GeneAmp PCR System 9700 (Applied Biosystems, Foster City, CA, USA) using a thermal cycler. Amplification products were used for sequence analysis; Purified using a gel extraction kit (QIAquick, Hilden, Germany). A total of 35 cycles of dideoxynucleotide sequencing (96 °C/10 s, 50 °C/5 s and 60 °C/6 min) were performed on both sides using the ABI Prism BigDye Terminator v3.1 (Applied Biosystems, Foster City, CA, USA). Sequence products were loaded into the ABI Prism 310 Genetic Analyzer (Applied Biosystems, Foster City, CA, USA) instrument and the obtained chromatograms were analyzed using the GenBank and the Basic Local Alignment Tool (BLAST) server in National Center for Biotechnology Information (NCBI). A total of 1200 nucleotide assays were performed and the isolate was identified as 100% *A. baumannii* based on the GenBank and the BLAST server in NCBI. In order to ensure the high level of accuracy in species-level identification of the sequences; *E*-value 0.0 and maximum identity ratios above 99% were used in the identification. The 100% value is the maximum identity rate.

Specific intrinsic blaOXA-51 (353 bp size) specific for *A. baumannii* species from OXA-type carbapenemases was investigated by in-house PCR (Polymerase Chain Reaction) method. For this purpose, DNA extraction of the strains was performed with a column-based DNA isolation kit (DNA mini kit, Qiagen, Hilden, Germany). GeneAmp PCR System 9700 (Applied Biosystems, Foster City, CA, USA) was used for the amplification. Amplification conditions; after initial denaturation at 94 °C for 3 min, denaturation at 94 °C for 30 s, a total of 35 cycles with primer binding at 60 °C for 30 s and 72 °C for 1 min, and then an elongation period for 10 min at 72 °C. The bands formed after 1.50% agarose gel electrophoresis of PCR products were evaluated with naked eyes with the help of UV transluminator and our isolate was PCR positive for blaOXA-51-like gene.

**Table:** 

Gene	Primer series	Product	Reference
blaOXA-51 group	F 5′-TAA TGC TTT GAT CGG CCT TG-3′ R 5′-TGG ATT GCA CTT CAT CTT GG-3′	353 base-pair	Woodford et al. (2006)

RT-PCR for Border disease virus and Peste des Petits Ruminants (PPR) as well as PCR for Caprine Herpes Virus-1 (CpHP-1) were performed in the paraffine sections from the liver, lung and brain. After deparaffinization, extraction was performed using viral nucleic acid extraction (GF-1 viral nucleic acid extraction kit, Vivantis, Selangor, Malaysia) as indicated by the manufacturer. For the Border disease virus, the nucleic acids that were extracuted before were reverse-transcribed and turned into complementary DNA. For this purpose, the procedure was performed using the fist strand cDNA synthesis kit (Thermo Fisher Scientific, Waltham, MA, USA) as recommended by manufacturer. The primers were used for the related diseases and Taq DNA polymerase enzyme treatment was performed. After the PCR, the samples were investigated in the sizes specified in the studies performed on the agarose gel. However, no evidence for the presence of border disease virus, PPR and CpHP-1 were found on PCR analysis (data not shown).

*A. baumannii* is a Gram negative, non-motile and ubiquitous aerobic coccobacillus causing oportunistic and nosocomial infections in humans. Hospital outbreaks have been emerging importance having a high mortality in humans for the last two decades. The predisposing factors are immune-compromising, prolonged length of hospital stay, extensive wounds, or having recently taken antibiotics (Francey et al. 2000; Peleg et al. 2008). However, the information concerning *A. baumannii* in veterinary medicine is very scarce (Müller et al. 2014). Most of the animal isolates were either clinical or environmental samples in the reports (Francey et al. 2000; Vaneechoutte et al. 2000; Endimiani et al. 2011; Eveillard et al. 2013; Guardabassi et al. 2004; Müller et al. 2014; Zordan et al. 2011), hence the relation between the agent and lesion was not well established. Moreover, *Acinetobacter* sp. has also been recognized as a normal microbial habitat in ruminant and dog gastro-intestinal system. Therefore, the significance of the isolation of this organism had always been unclear or undetermined in animals until the invasive infections due to *A. baumannii* were implicated in animal species (Brachelente et al. 2007; Jokisalo et al. 2010; Molenaar and van Engelen 2015).

It was stressed that *A. baumannii* might also be an emerging pathogen in veterinary medicine with a high potential for multidrug resistance and epidemics with unpredictable epidemiological risks (Endimiani et al. 2011; Müller et al. 2014; Zordan et al. 2011). However, there is still ongoing discussion on cross transmission between animals and humans (Guardabassi et al. 2004; Zordan et al. 2011). Although *A. baumannii* was reportedly responsible for fascitis in a cat (Brachelente et al. 2007), sepsis in a foal (Bentz et al. 2002), bronchopneumonia in minks (Molenaar and van Engelene 2015), pneumonia and sepsis in a pig (Zhang et al. 2013), dermatitis in falcons (Muller et al. 2010), and bronchopneumonia in a horse (Jokisalo et al. 2010), the importance of *A. baumannii* in animal infections has not been well-established. There is not any published report describing brain abscess and purulent bronchopneumonia due to *A. baumannii* neither in any animal species nor in sheep. In humans, brain abscess was diagnosed in a soldier who had been injured by shrapnel fragments in the head, face, thorax, and extremities (Guinand Vives et al. 2009). However, ther was no traumatic injury in the present case in history and macroscopically. This report clearly provides pathological and microbiological findings in a sheep due to *A. baumannii* and further indicates the pathogenic potential of this organism in livestock.

There is an important limitation to discuss epidemiologic considerations or evaluations depending on only one ovine case in the present report. However, the present report might mark the beginning of a new understanding of the importance of *A. baumannii* as a veterinary pathogen and it might give important stimuli for future studies. *A. baumannii* was regarded and as an emerging pathogen in veterinary medicine with a high potential for multidrug resistance and unpredictable epidemiological risks and it was further assumed that the livestock might be also the source of infection for man (Müller et al. 2014). These valuable considerations were supported on earlier published reports in which 16 different *A. baumannii* strains were isolated from pigs (8 strains) and cattle (8 strains) slaughtered for human consumption (Hamouda et al. 2011) and the isolation of the agent in a pig with pneumonia and sepsis (Zhang et al. 2013).

Overall, awareness of this pathogen as a potential agent in a ruminant species might be important for diagnosis of future cases in veterinary medicine and perhaps better understanding of epidemiology of the disease. Further studies are required to clarify the mechanisms involved in the pathogenesis and identify the predisposing factors, along with a complete characterization and epidemiology of the strains involved in animals and humans.
